# Deriving and interpreting population size estimates for adolescent and young key populations at higher risk of HIV transmission: Men who have sex with men and females who sell sex

**DOI:** 10.1371/journal.pone.0269780

**Published:** 2022-09-14

**Authors:** Lisa Grazina Johnston, Van Kinh Nguyen, Sudha Balakrishnan, Chibwe Lwamba, Aleya Khalifa, Keith Sabin

**Affiliations:** 1 Independent Consultant, UNICEF, New York, New York, United States of America; 2 MRC Centre for Global Infectious Disease Analysis, School of Public Health, Imperial College, London, United Kingdom; 3 UNICEF, New York, New York, United States of America; 4 UNAIDS, Geneva, Switzerland; University of Westminster, UNITED KINGDOM

## Abstract

Population sizes of adolescent (15- to 19-years) and young (20 to 24-years) key populations at risk for HIV transmission are essential for developing effective national HIV control strategies. We present new population size estimates of adolescent and young men who have sex with men and females who sell sex from 184 countries in nine UNICEF regions using UNAIDS published population size estimations submitted by national governments to derive 15-24-year-old population proportions based on the size of equivalent adult general populations. Imputed sizes based on regional estimates were used for countries or regions where adult proportion estimates were unavailable. Proportions were apportioned to adolescents and young adults based on age at sexual debut, by adjusting for the cumulative percentage of the sexually active population at each age for sex. Among roughly 69.5 million men who have sex with men, 12 million are under the age of 24 years, of whom 3 million are adolescents. There are an estimated 1.4 million adolescent and 3.7 million young females who sell sex. Roughly four and a half million adolescent men who have sex with men and females who sell sex would benefit from early HIV interventions. These population size estimates suggest there are roughly 17 million adolescent and young men who have sex with men and females who sell sex who need HIV prevention services and social support. These data provide evidence for national and international programs to determine how many adolescent and young key populations need essential health services and are living with HIV and other infections. Age disaggregated population sizes inform epidemic models, which increasingly use age-sex structures and are often used to obtain and allocate resources and human capacity and to plan critical prevention, treatment, and infection control programs.

## Introduction

The world has pledged to end AIDS as a public health threat by 2030. Although there has been notable progress in the past decade to end AIDS, key populations at higher risk of HIV, such as men who have sex with men and females who sell sex, make up about one-third of all new HIV infections—an estimated 450,000. They live with substantially higher risk of HIV transmission compared to the remaining population [1, 2]. A 2020 report by UNAIDS found that the risk of contracting HIV among men who have sex with men is 26 times higher compared to heterosexual men (aged 15 to 49) and for females who sell sex is 30 times higher compared to adult women [1]. Men who have sex with men and females who sell sex are less likely to seek vital HIV and other related health care due to societal stigma and discrimination, increased sexual, physical and emotional violence, and laws and policies that criminalize their behaviors [1, 3]. Among men who have sex with men and females who sell sex, adolescents (15- to 19-year-old) and young people (20- to 24-year-old) typically have less resilience, access and ability to protect themselves from HIV and other harmful health events [3, 4]. However, even though adolescent key populations engage in high-risk behaviors, they face significant legal obstacles to obtaining these essential and lifesaving services because of their ages. In most countries, adolescents are legally restricted from obtaining HIV testing, counselling and treatment without parental consent–this can be a significant structural and policy barrier to adolescents who engage in same sex relationships or sell sex and who may not want to disclose their behaviors to a parent or do not have any legal guardian [4, 5]. They face these obstacles as minors, often without family support. Without major improvements and scale-up in HIV prevention, testing and treatment programs that focus on adolescent and young people’s unique circumstances and needs, an additional 379,000 children and adolescents are projected to die of AIDS-related diseases between 2017 and 2030 [6, 7].

The sizes of populations at risk for HIV transmission are an essential component for effective national and international HIV control strategies. Population sizes inform epidemic models, which increasingly use age-sex structures [8] and are used to obtain and allocate adequate resources and human capacity for critical prevention and infection control programs. An understanding of the scope and size of populations affected by HIV is the foundation of an effective HIV response. Adolescent and young key populations require a discrete set of services that will differ from those needed and used by adults. Adolescent and young key populations, like their adult peers need access to commodities such as condoms, lubricants, female-controlled contraceptives, pre-exposure prophylaxes and HIV testing. They will also typically need introduction to sexual and reproductive health, comprehensive sexual education, mental health interventions, and parental and peer support [9]. Community organizations can be critical in delivering these services but they also need to plan and mobilize adequate resources. Furthermore, adolescents and young people require different outreach methods to improve access to HIV and other health services, such as youth-friendly programs and digital platforms [10].

Over the past decade there has been substantial progress in estimating the population sizes of adult men who have sex with men and females who sell sex [11]; however, there are no reliable data on those who are adolescents or young. Population size estimations of adult men who have sex with men and females who sell sex are derived mostly from methods incorporated into Integrated Biological and Behavioral Surveillance (IBBS) surveys using probability-based sampling methods, such as respondent driven sampling (RDS) or time location sampling (TLS) [11]. The population size methods most often used with IBBS are the unique object and service multipliers [12–14], wisdom of the crowds [15], capture/recapture (overlapping of two probability-based surveys), and the successive sampling population size estimation (SS-PSE) [16, 17]. Other methods used to estimate the size of hidden populations include mapping and enumeration [18–20], PLACE [21], multiple source capture-recapture [22] and network scale up [23–25].

This paper presents new estimates of the population sizes of adolescent and young men who have sex with men and females who sell sex to build a foundation upon which effective action can be taken to address the HIV epidemic. In addition, the most up to date prevalence of HIV is presented to show the magnitude of infection among young key populations.

## Methods

Country population size estimates and HIV prevalence data are based on data submitted between 2014 and 2019 by national governments to UNAIDS through the Global AIDS Monitoring system. Data submitted to UNAIDS are displayed in AIDSInfo (aidsinfo.unaids.org). These data are supplemented in the key population atlas (http://www.aidsinfoonline.org/kpatlas/#/home) which includes other estimates that are published in reports and peer-reviewed journals. HIV prevalence data are mostly based on national HIV biological behavioral surveillance survey findings.

The size estimates submitted to UNAIDS are evaluated regarding national representativeness based on a number of factors including the methodologies used to estimate population sizes, the percentage of the population included in the estimations and the methods used to conduct an extrapolation. Estimates that are deemed “subnational” are extrapolated here to reflect a national estimate. The extrapolation of “subnational” estimates uses the UNAIDS regional median population proportion for all reported size estimates multiplied by the adult population (aged 15–49) of the relevant sex (i.e., males for men who have sex with men and females who sell sex). The regional medians are published in Table 1 in the Spectrum Quickstart guide (https://www.unaids.org/sites/default/files/media_asset/QuickStartGuide_Spectrum_en.pdf). The male and female 15–49 populations are drawn from the World Population Prospects (WPP): 2019 Revision. (https://population.un.org/wpp/). In countries or regions where key population proportion estimates in adults were not available, median proportions of the region were imputed, upon which a spatial smoothing was done. In particular, the logit of the proportion was regressed against a global mean of the population size proportion, a varying mean for each of the UNICEF defined subregions formulated as a random effect (26), and a weighted average of countries that share borders formulated as an Intrinsic Conditional Auto-Regressive term (ICAR). The smoothing was done using integrated nested Laplace approximations (INLA) package (1). Based on the calculated proportion and the national population aged 15–49 obtained from World Population Prospects, PSE for each country aged 15–49 (*PSE*_15:49_) was obtained. This number was then apportioned to adolescents and young adults based on age at sexual debut, by adjusting for the cumulative percentage of the sexually active population at each age *a* and sex *s (ρ*_*sa*_*)*, using data from the Demographic and Health Surveys (DHS), Multiple Indicator Cluster Surveys (MICS), Health Behaviour in School-aged Children (HBSC), and National Survey of Family Growth (NSFG). The sexual debut rate was estimated separately from the prevalence model. In particular, we used a survival model in which the time to the age at first sex was assumed following a log-logistic distribution. Individuals who had never had sex at the time of the interview were considered “right censored” based on the age at the time of the interview. The log of time since birth to age at first sex was then regressed against a global intercept, a region-specific intercept defined by UNICEF, and a neighboring ICAR structure model. The survey weights were scaled to the sample size and incorporated into the likelihood(1). Once the sexual debut distribution by country and sex were calculated, the PSE for age *a* and sex *s* was apportioned as $PSE_{sa}=N_{sa}\;\rho_{sa}\frac{PSE_{15:49}}{\sum_{a}N_{sa}\rho_{sa}}$, where *N*_*sa*_ is the total population size age *a* obtained from WPP2019 and *s* is male (for men who have sex with men and transgender women) and female (for females who sell sex) is the total population size age *a* obtained from WPP2019. The source code for the main steps in the smoothing, the sexual debut rate estimate, and the model constraints specifications for can be found at https://github.com/kklot/KPsize. For the purposes of this paper, men who have sex with men can be defined as having anal sex with a man in the past six months or year, females who sell sex can be defined as having exchanged sex for goods or money in the past six months or year.

## Results

Population size estimations were calculated for men who have sex with men and females who sell sex in 184 countries. Table 1 shows the population size estimates by UNICEF region.

**Table 1:** Population sizes of men who have sex with men and females who sell sex, by age and UNICEF region.

	Men who have sex with men			Females who sell sex		
	15–19	20–24	25–49	15–19	20–24	25–49
**East Asia and the Pacific**	439,000	1,856,000	15,684,000	163,000	696,000	6,072,000
**Eastern Europe and central Asia**	106,000	451,000	4,470,000	32,000	135,000	1,413,000
**East and southern Africa**	202,000	406,000	1,442,000	343,000	694,000	2,535,000
**Latin America and the Caribbean**	375,000	1,253,000	7,303,000	137,000	453,000	2,713,000
**Middle East and North Africa**	56,000	218,000	1,742,000	50,000	191,000	1,438,000
**North America**	353,000	931,000	5,492,000	53,000	140,000	846,000
**South Asia**	690,000	2,092,000	10,930,000	249,000	753,000	4,066,000
**West and central Africa**	449,000	804,000	2,747,000	289,000	517,000	1,787,000
**West and central Europe**	322,000	993,000	7,642,000	45,000	140,000	1,124,000

Tables 2 and 3, list the regions, countries, size estimations by age groups and percentage of the key population by age groups, percent of equivalent general population by sex and age group and HIV prevalence for those countries that reported it. The UNICEF regions consist of East Asia and Pacific (EAP), Eastern Europe and Central Asia (EECA), East and South Africa (ESA), Latin America and the Caribbean (LAC), Middle East and North Africa (MENA), North America (NA), South Asia (SA), West and Central Africa (WCA) and West and Central Europe (WE).

**Table 2:** Population size estimations for adolescent and young men who have sex with men, 2019.

Region	Country	Size estimate by age groups (counts)				Percent of all men who have sex with men by age groups			Percent of equivalent male general population by age group			HIV prevalence
		15–19	20–24	15–24	25–49	15–19	20–24	25–49	15–19	20–24	25–49	<25
EAP	China	236530	1054650	1291170	9744730	2.14	9.56	88.3	0.06	0.29	2.66	5.6 (2018)
EAP	Indonesia*	86760	286950	373710	1656730	4.27	14.13	81.59	0.12	0.39	2.22	23.8 (2015)
EAP	Japan	27170	93610	120780	790350	2.98	10.27	86.74	0.11	0.37	3.1	2.8 (2015)
EAP	Philippines*	16490	95380	111860	714750	1.99	11.54	86.47	0.06	0.32	2.39	3.7 (2018)
EAP	Thailand*	14480	61110	75590	442240	2.8	11.8	85.4	0.09	0.36	2.62	6.2 (2018)
EAP	Australia*	11520	33140	44660	225190	4.27	12.28	83.45	0.19	0.55	3.75	1.2 (2014)
EAP	Malaysia	8490	32860	41350	206030	3.43	13.28	83.28	0.09	0.35	2.18	15.5 (2017)
EAP	South Korea	6990	37640	44630	337730	1.83	9.84	88.33	0.06	0.3	2.69	4.3 (2011)
EAP	Vietnam	6720	48420	55150	692370	0.9	6.48	92.62	0.03	0.18	2.63	10.6 (2018)
EAP	Myanmar*	5340	35280	40610	361090	1.33	8.78	89.89	0.04	0.24	2.47	4.2 (2018)
EAP	North Korea	5240	22640	27890	162040	2.76	11.92	85.32	0.08	0.34	2.45	NA
EAP	Cambodia	3610	18500	22110	120240	2.53	13	84.47	0.08	0.41	2.69	0.6 (2015)
EAP	Papua New Guinea*	3400	11180	14590	52750	5.05	16.61	78.34	0.14	0.46	2.17	NA
EAP	New Zealand	2390	7100	9490	40880	4.74	14.1	81.16	0.22	0.65	3.76	NA
EAP	Laos	1160	6590	7750	48320	2.07	11.75	86.18	0.06	0.33	2.39	2.1 (2017)
EAP	Mongolia†	590	2520	3110	21320	2.4	10.32	87.28	0.07	0.29	2.48	0.7 (2017)
EAP	Singapore	500	4100	4590	41410	1.08	8.9	90.02	0.03	0.27	2.69	23.8 (2015)
EAP	Timor-Leste	330	1520	1850	7960	3.39	15.46	81.15	0.1	0.44	2.32	NA
EAP	Solomon Islands	240	880	1120	4130	4.61	16.78	78.61	0.14	0.51	2.39	NA
EAP	Fiji	230	880	1110	5720	3.32	12.89	83.78	0.1	0.37	2.4	NA
EAP	Brunei	100	400	500	2850	3.07	11.93	85	0.08	0.31	2.19	NA
EAP	Vanuatu	100	360	460	1880	4.37	15.4	80.22	0.13	0.47	2.43	NA
EAP	Samoa*	<100	270	360	1180	5.9	17.24	76.86	0.18	0.53	2.38	0 (2018)
EAP	Tonga	<100	160	210	580	6.88	19.88	73.23	0.21	0.61	2.23	NA
EAP	Micronesia (Federated States of) †	<100	160	200	670	5.31	17.8	76.89	0.15	0.49	2.12	NA
EAP	Kiribati†	<100	150	200	680	4.95	17.29	77.76	0.15	0.52	2.32	NA
EECA	Russia	41670	134360	176020	1468590	2.53	8.17	89.3	0.12	0.4	4.38	NA
EECA	Turkey	16560	101280	117840	952720	1.55	9.46	88.99	0.07	0.45	4.25	NA
EECA	Ukraine*	9800	41160	50950	456430	1.93	8.11	89.96	0.1	0.4	4.46	6.7 (2017)
EECA	Romania	7490	29270	36770	273240	2.42	9.44	88.14	0.17	0.66	6.17	11.6 (2011)
EECA	Uzbekistan	6880	34620	41500	260180	2.28	11.47	86.24	0.08	0.38	2.84	2.9 (2018)
EECA	Bulgaria	3730	9920	13650	99180	3.31	8.79	87.9	0.24	0.64	6.41	1.3 (2016)
EECA	Serbia	3020	15140	18160	144070	1.86	9.33	88.81	0.15	0.74	7	2.8 (2013)
EECA	Kazakhstan	2820	13960	16780	149320	1.7	8.4	89.9	0.06	0.31	3.31	6.7 (2018)
EECA	Tajikistan	2340	11680	14010	72960	2.68	13.43	83.89	0.1	0.48	3	1.7 (2017)
EECA	Belarus*	2240	8120	10360	96540	2.1	7.59	90.31	0.1	0.37	4.45	4.0 (2013)
EECA	Croatia	1480	7630	9110	66140	1.97	10.14	87.9	0.17	0.85	7.41	1.5 (2013)
EECA	Turkmenistan	1280	5760	7040	46200	2.4	10.82	86.78	0.08	0.36	2.9	NA
EECA	Kyrgyzstan†	1260	6540	7800	50700	2.16	11.18	86.66	0.08	0.39	3.03	5.7 (2017)
EECA	Bosnia & Herzegovina	1010	6470	7480	53470	1.66	10.61	87.72	0.14	0.87	7.15	NA
EECA	Azerbaijan*	1010	7160	8170	85910	1.07	7.61	91.32	0.04	0.27	3.2	0.8 (2018)
EECA	Albania	960	5840	6800	48500	1.73	10.57	87.7	0.13	0.81	6.7	NA
EECA	Moldova*	740	3730	4470	41400	1.61	8.13	90.26	0.07	0.35	3.9	7.3 (2017)
EECA	Georgia*	430	2800	3220	32480	1.19	7.83	90.98	0.05	0.31	3.59	8.8 (2018)
EECA	Armenia*	370	2230	2590	25530	1.3	7.91	90.78	0.05	0.32	3.67	0.6 (2018)
EECA	Macedonia*	350	2540	2890	36480	0.9	6.45	92.65	0.07	0.48	6.96	4.4 (2017)
EECA	Montenegro	230	1180	1410	10370	1.92	10.02	88.06	0.15	0.78	6.88	3.6 (2014)
ESA	Ethiopia	38370	82560	120930	265910	9.92	21.34	68.74	0.13	0.28	0.89	NA
ESA	Angola	22480	35290	57770	113080	13.16	20.66	66.19	0.29	0.46	1.48	8.2 (2011)
ESA	Tanzania	19480	38690	58160	141070	9.78	19.42	70.81	0.13	0.26	0.96	15.4 (2014)
ESA	Uganda	18960	33270	52220	100090	12.45	21.84	65.71	0.17	0.31	0.92	NA
ESA	Kenya	16750	34930	51680	128320	9.31	19.41	71.29	0.12	0.24	0.89	12.2 (2011)
ESA	Mozambique	13840	21130	34970	63190	14.1	21.53	64.37	0.19	0.28	0.85	NA
ESA	Madagascar	12070	23640	35700	80370	10.4	20.36	69.24	0.17	0.34	1.14	9.0 (2014)
ESA	South Africa*	11640	30790	42420	172250	5.42	14.34	80.24	0.07	0.19	1.05	0 (2018)
ESA	Sudan	10070	27520	37590	103540	7.13	19.5	73.36	0.09	0.25	0.94	0.8 (2015)
ESA	Malawi*	7400	13180	20580	41730	11.88	21.15	66.97	0.16	0.28	0.88	NA
ESA	Zambia†	7060	12290	19350	39120	12.07	21.02	66.9	0.15	0.27	0.86	NA
ESA	Somalia	5710	11590	17310	32490	11.47	23.28	65.24	0.16	0.31	0.88	NA
ESA	Zimbabwe†	5560	9590	15150	30010	12.32	21.23	66.45	0.16	0.27	0.85	NA
ESA	South Sudan	3830	7780	11610	26370	10.09	20.48	69.44	0.14	0.28	0.94	NA
ESA	Rwanda	3420	8060	11470	31530	7.94	18.74	73.32	0.1	0.25	0.97	2.3 (2016)
ESA	Burundi	1850	6600	8450	31400	4.64	16.56	78.8	0.06	0.23	1.1	1.1 (2011)
ESA	Eritrea	1190	2110	3300	8680	9.93	17.61	72.45	0.14	0.24	0.99	NA
ESA	Botswana†	520	1360	1880	6520	6.21	16.16	77.63	0.08	0.22	1.04	NA
ESA	Namibia	500	1570	2070	6950	5.52	17.43	77.05	0.08	0.24	1.05	NA
ESA	Lesotho†	450	1260	1710	5880	5.94	16.59	77.47	0.07	0.21	0.98	NA
ESA	Swaziland†	360	840	1200	2980	8.64	20.07	71.28	0.12	0.27	0.97	NA
ESA	Mauritius	250	700	950	4320	4.69	13.34	81.97	0.08	0.21	1.32	5.5 (2015)
ESA	Djibouti	230	530	760	2500	7.1	16.34	76.57	0.08	0.18	0.84	NA
ESA	Comoros	150	570	720	2970	3.94	15.48	80.58	0.06	0.25	1.31	0 (2018)
ESA	Seychelles	<100	<100	<100	360	3.93	11.25	84.82	0.06	0.18	1.38	0.6 (2013)
LAC	Brazil	114480	391500	505970	2400290	3.94	13.47	82.59	0.2	0.68	4.19	NA
LAC	Mexico*	55220	230170	285390	1447490	3.19	13.28	83.53	0.16	0.68	4.27	11.9 (2013)
LAC	Colombia	34970	105540	140510	550230	5.06	15.28	79.66	0.25	0.77	4.01	9.5 (2016)
LAC	Argentina	25420	83390	108820	495760	4.2	13.79	82	0.22	0.73	4.32	NA
LAC	Venezuela	22340	56360	78700	311450	5.73	14.44	79.83	0.31	0.79	4.37	NA
LAC	Guatemala	15070	44260	59320	180300	6.29	18.47	75.24	0.31	0.91	3.7	6.4 (2017)
LAC	Peru	13830	51730	65560	383990	3.08	11.51	85.42	0.16	0.59	4.4	14.5 (2018)
LAC	Haiti†	13630	33320	46960	142780	7.19	17.56	75.25	0.45	1.1	4.69	13.3 (2011)
LAC	Dominican Republic*	10340	29060	39400	143320	5.66	15.9	78.44	0.36	1.01	4.98	NA
LAC	Ecuador	9530	35710	45240	196400	3.94	14.78	81.28	0.2	0.76	4.15	NA
LAC	Bolivia	8710	26560	35270	125360	5.42	16.54	78.04	0.28	0.85	4.02	NA
LAC	Chile	8260	31280	39540	218110	3.2	12.14	84.66	0.17	0.63	4.42	9.7 (2016)
LAC	Cuba*	8150	24270	32420	158270	4.27	12.73	83	0.31	0.94	6.12	0.8 (2018)
LAC	Honduras	8070	23930	32000	101690	6.03	17.9	76.07	0.29	0.85	3.62	8.2 (2018)
LAC	Nicaragua	5240	14080	19320	68530	5.96	16.03	78.01	0.29	0.77	3.77	6.3 (2016)
LAC	El Salvador*	4340	14840	19180	62930	5.28	18.08	76.64	0.27	0.92	3.89	8.9 (2018)
LAC	Paraguay*	4240	15510	19750	79680	4.26	15.6	80.14	0.21	0.78	4.01	12.7 (2017)
LAC	Costa Rica†	2800	9210	12000	56140	4.1	13.51	82.39	0.21	0.68	4.15	NA
LAC	Jamaica*	2690	8030	10730	39110	5.41	16.12	78.47	0.35	1.03	5.01	19.2 (2018)
LAC	Panama†	2560	8230	10790	46490	4.47	14.37	81.16	0.23	0.73	4.13	5.7 (2018)
LAC	Uruguay	1840	6250	8090	37960	4	13.57	82.44	0.22	0.74	4.49	5.5 (2013)
LAC	Guyana	760	2330	3090	9250	6.16	18.9	74.94	0.37	1.13	4.46	4.4 (2014)
LAC	Trinidad & Tobago	680	2370	3060	20370	2.92	10.12	86.96	0.19	0.67	5.73	NA
LAC	Suriname	450	1400	1850	7380	4.9	15.14	79.96	0.29	0.9	4.75	13.9 (2018)
LAC	Bahamas†	350	990	1340	5150	5.38	15.23	79.39	0.34	0.95	4.95	NA
LAC	Barbados	240	650	890	3830	5.08	13.69	81.23	0.36	0.98	5.81	11.3 (2014)
LAC	Belize	240	870	1110	4280	4.44	16.09	79.47	0.22	0.79	3.89	10.5 (2012)
LAC	St. Lucia*	140	450	590	2480	4.51	14.6	80.89	0.28	0.91	5.04	NA
LAC	St. Vincent & Grenadines	110	300	410	1500	5.62	15.78	78.6	0.37	1.04	5.17	NA
LAC	Grenada	<100	260	350	1570	4.44	13.76	81.8	0.29	0.91	5.4	NA
LAC	Antigua & Barbuda	<100	240	320	1280	5.04	14.88	80.08	0.33	0.99	5.32	NA
MENA	Iran*	15530	47110	62640	387800	3.45	10.46	86.09	0.07	0.2	1.66	NA
MENA	Egypt	11160	45320	56480	277870	3.34	13.55	83.11	0.04	0.17	1.05	6.6 (2017)
MENA	Morocco*	6410	16940	23350	90980	5.61	14.81	79.58	0.07	0.18	0.96	4.2 (2017)
MENA	Algeria	6390	17640	24020	119210	4.46	12.31	83.23	0.06	0.16	1.05	3.3 (2017)
MENA	Iraq	4380	25350	29730	184220	2.05	11.85	86.1	0.04	0.23	1.7	NA
MENA	Yemen	2250	13030	15280	92180	2.09	12.13	85.78	0.03	0.16	1.14	3.1 (2011)
MENA	Tunisia	1640	4810	6450	30580	4.44	12.98	82.58	0.05	0.16	1.02	10.6 (2014)
MENA	Syria	1530	9950	11480	85420	1.58	10.27	88.16	0.03	0.2	1.75	NA
MENA	Israel	1490	9120	10610	88480	1.5	9.2	89.3	0.07	0.44	4.29	NA
MENA	Saudi Arabia	1220	8240	9460	151360	0.76	5.12	94.12	0.01	0.07	1.23	NA
MENA	Libya	1070	2990	4060	19040	4.62	12.96	82.41	0.05	0.15	0.96	NA
MENA	Jordan	960	5490	6450	44880	1.87	10.7	87.44	0.03	0.19	1.58	NA
MENA	Palestinian Territories	590	3410	3990	22620	2.2	12.8	85	0.04	0.25	1.67	NA
MENA	Lebanon*	540	3490	4030	31380	1.52	9.86	88.62	0.03	0.2	1.76	NA
MENA	Kuwait	140	810	960	20910	0.65	3.72	95.63	0.01	0.05	1.35	NA
MENA	United Arab Emirates	140	2000	2140	46750	0.29	4.09	95.62	0	0.04	0.9	NA
MENA	Bahrain	<100	420	510	10810	0.8	3.67	95.53	0.01	0.05	1.35	NA
MENA	Oman	<100	820	890	23210	0.29	3.41	96.3	0	0.03	0.95	NA
MENA	Qatar	<100	780	820	14670	0.26	5.01	94.74	0	0.05	0.88	NA
NA	United States	328120	845200	1173320	4900390	5.4	13.92	80.68	0.43	1.1	6.36	NA
NA	Canada	24850	85350	110200	591200	3.54	12.17	84.29	0.29	0.99	6.85	2.1 (2011)
SA	India	442120	1503840	1945950	8332960	4.3	14.63	81.07	0.11	0.38	2.09	3.4 (2015)
SA	Bangladesh*	124950	204220	329170	866380	10.45	17.08	72.47	0.27	0.43	1.84	0 (2015)
SA	Pakistan*	69520	257140	326660	1279000	4.33	16.01	79.66	0.12	0.43	2.13	3.6 (2016)
SA	Afghanistan†	24580	58730	83310	197790	8.74	20.89	70.36	0.24	0.57	1.92	0 (2012)
SA	Nepal*	18860	44730	63590	128770	9.8	23.25	66.94	0.27	0.63	1.82	5.3 (2017)
SA	Sri Lanka*	10190	22240	32430	115590	6.88	15.03	78.09	0.2	0.45	2.32	0 (2016)
SA	Bhutan	230	830	1060	4850	3.9	14.04	82.05	0.09	0.34	1.97	NA
SA	Maldives	<100	440	490	4460	0.87	8.99	90.15	0.02	0.17	1.75	NA
WCA	Nigeria*	153970	301920	455890	1084870	9.99	19.6	70.41	0.3	0.6	2.14	18.6 (2015)
WCA	Congo-Kinshasa*	76330	127070	203400	412190	12.4	20.64	66.96	0.37	0.61	1.98	2.1 (2016)
WCA	Niger	30660	40750	71410	107590	17.13	22.77	60.1	0.58	0.77	2.03	NA
WCA	Côte d’Ivoire	24390	40320	64710	130260	12.51	20.68	66.81	0.37	0.61	1.96	9.5 (2015)
WCA	Cameroon*	22710	36700	59410	127420	12.16	19.64	68.2	0.34	0.55	1.9	28.8 (2011)
WCA	Mali†	21970	31800	53770	96150	14.65	21.21	64.14	0.46	0.67	2.02	10.5 (2015)
WCA	Ghana*	18570	42180	60750	169670	8.06	18.31	73.64	0.23	0.51	2.06	NA
WCA	Chad	16770	25120	41880	72680	14.63	21.92	63.44	0.43	0.64	1.86	NA
WCA	Burkina Faso	16080	31750	47830	105320	10.5	20.73	68.77	0.31	0.62	2.05	1.2 (2017)
WCA	Guinea	13180	21650	34830	58440	14.13	23.21	62.66	0.42	0.69	1.85	11.4 (2017)
WCA	Benin	8930	18000	26930	61850	10.06	20.27	69.67	0.3	0.6	2.07	10.2 (2017)
WCA	Senegal	7650	22870	30530	90460	6.33	18.91	74.77	0.19	0.57	2.26	19.1 (2018)
WCA	Sierra Leone	7330	11530	18870	39370	12.59	19.81	67.6	0.35	0.56	1.9	5.7 (2011)
WCA	Togo*	6060	11530	17590	42680	10.06	19.13	70.81	0.29	0.55	2.03	14.6 (2017)
WCA	Central African Republic	5360	7720	13090	20350	16.04	23.1	60.85	0.45	0.65	1.71	5.4 (2017)
WCA	Liberia*	5170	7440	12610	24590	13.9	20.01	66.1	0.4	0.58	1.91	NA
WCA	Congo-Brazzaville	4860	7260	12120	26140	12.71	18.96	68.32	0.35	0.53	1.9	32.2 (2018)
WCA	Mauritania	3680	7350	11030	28600	9.27	18.55	72.17	0.31	0.61	2.39	NA
WCA	Gabon	1450	2460	3910	12130	9.02	15.34	75.65	0.24	0.41	2.02	NA
WCA	Guinea-Bissau*	1270	2760	4030	10110	8.97	19.52	71.51	0.26	0.57	2.08	NA
WCA	Gambia†	1210	3460	4670	12940	6.89	19.63	73.48	0.21	0.59	2.22	35.5 (2018)
WCA	Equatorial Guinea	770	1820	2580	8520	6.89	16.34	76.77	0.16	0.39	1.82	NA
WCA	Cape Verde	250	620	860	3660	5.48	13.61	80.91	0.15	0.38	2.24	6.6 (2013)
WCA	São Tomé & Príncipe	180	330	510	1220	10.34	19.27	70.39	0.33	0.61	2.23	0.8 (2018)
WE	Germany	64460	191230	255690	1300120	4.14	12.29	83.57	0.37	1.08	7.37	1.1 (2016)
WE	United Kingdom	60890	150900	211790	953210	5.23	12.95	81.82	0.4	1	6.31	1.5 (2015)
WE	France	44970	144190	189170	1001250	3.78	12.11	84.11	0.33	1.05	7.32	1.5 (2011)
WE	Italy	38750	121290	160030	967770	3.44	10.75	85.81	0.31	0.97	7.76	NA
WE	Spain	16370	72170	88540	789060	1.87	8.22	89.91	0.16	0.7	7.61	7.2 (2015)
WE	Netherlands	15100	42070	57160	264870	4.69	13.06	82.25	0.41	1.13	7.14	NA
WE	Poland	10210	48070	58280	544840	1.69	7.97	90.34	0.11	0.53	6.05	1.6 (2014)
WE	Sweden	8820	21460	30280	157250	4.7	11.44	83.85	0.39	0.96	7.03	1.0 (2013)
WE	Belgium	8400	25540	33940	182700	3.88	11.79	84.33	0.33	1	7.15	0.5 (2015)
WE	Austria	7040	19820	26860	140160	4.21	11.87	83.92	0.35	0.99	6.98	NA
WE	Czechia	6570	15810	22380	158620	3.63	8.74	87.64	0.27	0.64	6.42	1.4 (2011)
WE	Portugal	5440	19610	25060	158690	2.96	10.67	86.36	0.26	0.92	7.46	2.8 (2011)
WE	Norway	5430	13550	18980	81730	5.39	13.46	81.15	0.42	1.05	6.35	NA
WE	Denmark	5290	15370	20660	89890	4.78	13.91	81.31	0.42	1.21	7.06	NA
WE	Hungary	4930	15650	20580	134580	3.17	10.09	86.74	0.22	0.69	5.97	4 (2011)
WE	Switzerland	4430	17030	21460	140200	2.74	10.53	86.73	0.23	0.89	7.29	3.8 (2013)
WE	Finland	3840	11940	15780	84660	3.82	11.89	84.29	0.32	1	7.08	NA
WE	Ireland*	2900	9360	12250	72890	3.4	10.99	85.61	0.25	0.8	6.26	2.5 (2016)
WE	Greece*	2640	14490	17120	173960	1.38	7.58	91.04	0.12	0.64	7.65	NA
WE	Slovakia	1390	6940	8330	81710	1.54	7.71	90.75	0.1	0.52	6.12	NA
WE	Slovenia	970	3520	4490	35050	2.44	8.91	88.64	0.21	0.77	7.67	NA
WE	Lithuania	890	4000	4890	41200	1.94	8.67	89.39	0.16	0.7	7.23	0 (2011)
WE	Latvia	850	2320	3160	28440	2.68	7.33	89.99	0.21	0.58	7.16	3.1 (2011)
WE	Estonia	600	1810	2410	20530	2.61	7.89	89.5	0.2	0.6	6.8	0 (2018)
WE	Cyprus	320	2030	2350	16350	1.71	10.84	87.45	0.1	0.64	5.17	NA
WE	Luxembourg	320	1270	1590	10350	2.68	10.6	86.72	0.2	0.8	6.52	NA
WE	Iceland	290	800	1090	5130	4.69	12.84	82.47	0.36	0.98	6.32	NA
WE	Malta	220	830	1040	6960	2.69	10.33	86.98	0.21	0.8	6.75	NA

*Based on adult data assessed as nationally adequate;

^†^ Based on adult data assessed as nationally inadequate but regionally adequate;

all other countries have either no documented size estimates and/or used inadequate methods.

**Table 3:** Population size estimations for adolescent and young females who sell sex, 2019.

		Size estimate by age groups (counts)				Percent of all females who sell sex by age groups			Percent of equivalent female general population by age group			HIV prevalence
EAP	Country	15–19	20–24	15–24	25–49	15–19	20–24	25–49	15–19	20–24	25–49	<25
EAP	China	78840	357520	436360	3515890	1.99	9.05	88.96	0.02	0.11	1.04	0.1 (2018)
EAP	Indonesia*	36000	119730	155730	720970	4.11	13.66	82.24	0.05	0.17	0.99	4.1 (2015)
EAP	Japan	10550	36380	46930	310200	2.95	10.19	86.86	0.04	0.15	1.27	NA
EAP	Philippines*	7460	43660	51120	340520	1.91	11.15	86.95	0.03	0.15	1.18	0.7 (2015)
EAP	Thailand*	7300	31110	38410	241160	2.61	11.13	86.26	0.04	0.18	1.42	2.8 (2017)
EAP	Australia	3800	12550	16340	59990	4.97	16.44	78.59	0.16	0.54	2.59	12.7 (2011)
EAP	Malaysia	3310	12840	16140	78890	3.48	13.51	83.01	0.04	0.15	0.90	0 (2017)
EAP	South Korea	2850	8300	11150	58000	4.12	12.00	83.88	0.05	0.14	0.98	0 (2013)
EAP	Vietnam	2590	13850	16440	124770	1.83	9.81	88.36	0.02	0.12	1.08	NA
EAP	Myanmar*	2490	18190	20680	269390	0.86	6.27	92.87	0.01	0.07	1.06	1.6 (2017)
EAP	North Korea	2120	10680	12790	74190	2.43	12.28	85.29	0.05	0.23	1.62	0.7 (2016)
EAP	Cambodia	2060	13780	15830	148400	1.25	8.39	90.36	0.01	0.09	0.97	3.7 (2018)
EAP	Papua New Guinea*	1990	8560	10550	62650	2.72	11.69	85.59	0.03	0.13	0.97	NA
EAP	New Zealand	590	1750	2340	10910	4.48	13.18	82.34	0.05	0.16	0.99	NA
EAP	Laos	570	3240	3810	24060	2.03	11.63	86.33	0.03	0.16	1.21	1 (2017)
EAP	Mongolia†	220	940	1160	8110	2.35	10.16	87.49	0.03	0.11	0.94	0 (2017)
EAP	Singapore	180	1280	1460	14120	1.15	8.20	90.66	0.01	0.09	1.03	NA
EAP	Timor-Leste	140	610	750	3240	3.38	15.36	81.26	0.04	0.18	0.97	NA
EAP	Solomon Islands	<100	330	420	1700	4.35	15.58	80.07	0.05	0.20	1.00	0 (2017)
EAP	Fiji	<100	360	450	2330	3.29	12.97	83.73	0.04	0.16	1.03	0 (2015)
EAP	Brunei	<100	150	190	1040	3.24	12.01	84.75	0.03	0.13	0.88	NA
EAP	Vanuatu	<100	130	170	780	3.89	14.15	81.96	0.05	0.17	1.00	NA
EAP	Samoa*	<100	<100	120	410	6.10	17.14	76.76	0.07	0.20	0.91	0 (2018)
EAP	Tonga	<100	<100	<100	230	6.11	17.45	76.44	0.07	0.19	0.85	NA
EAP	Micronesia (Federated States of)†	<100	<100	<100	240	5.30	17.78	76.92	0.06	0.19	0.81	NA
EAP	Kiribati†	<100	<100	<100	270	4.56	16.43	79.01	0.05	0.18	0.88	NA
EECA	Russia	13960	45230	59190	525540	2.39	7.74	89.88	0.04	0.13	1.56	NA
EECA	Turkey	3790	23360	27160	228740	1.48	9.13	89.39	0.02	0.11	1.03	NA
EECA	Ukraine*	3180	13490	16670	157870	1.82	7.73	90.45	0.03	0.13	1.56	1.3 (2017)
EECA	Romania	2950	14920	17870	116590	2.20	11.10	86.71	0.03	0.17	1.29	1.7 (2018)
EECA	Uzbekistan	1320	5090	6410	47870	2.43	9.38	88.19	0.03	0.12	1.15	1.4 (2011)
EECA	Bulgaria	1240	6090	7330	70140	1.60	7.86	90.54	0.03	0.13	1.55	0.6 (2017)
EECA	Serbia	970	4920	5890	32060	2.57	12.95	84.48	0.04	0.21	1.34	2.5 (2018)
EECA	Kazakhstan*	750	2730	3480	34150	2.00	7.25	90.75	0.04	0.13	1.60	3.8 (2017)
EECA	Tajikistan*	650	1710	2360	17170	3.31	8.76	87.93	0.04	0.12	1.19	0 (2016)
EECA	Belarus*	570	2580	3150	21420	2.33	10.48	87.19	0.04	0.16	1.34	NA
EECA	Croatia	550	2860	3410	23040	2.08	10.81	87.11	0.03	0.17	1.39	0.9 (2016)
EECA	Turkmenistan	480	3540	4020	48090	0.92	6.80	92.28	0.02	0.14	1.84	4.1 (2018)
EECA	Kyrgyzstan	400	2020	2430	20000	1.80	9.02	89.18	0.02	0.10	1.01	0 (2013)
EECA	Bosnia & Herzegovina	240	1240	1470	14190	1.52	7.89	90.59	0.02	0.12	1.36	0 (2017)
EECA	Azerbaijan*	210	1060	1270	9420	1.92	9.95	88.13	0.02	0.12	1.10	NA
EECA	Albania	170	1130	1310	15170	1.04	6.88	92.08	0.02	0.13	1.70	0 (2017)
EECA	Moldova*	140	870	1010	7400	1.67	10.38	87.95	0.02	0.12	1.03	NA
EECA	Georgia	130	770	900	11510	1.01	6.21	92.78	0.02	0.10	1.54	0 (2018)
EECA	Armenia*	120	770	890	6220	1.65	10.88	87.46	0.02	0.12	0.94	NA
EECA	Macedonia	<100	330	380	4860	0.89	6.36	92.75	0.01	0.07	0.97	0 (2018)
EECA	Montenegro	<101	150	180	1420	1.79	9.47	88.74	0.02	0.10	0.98	0 (2015)
ESA	Ethiopia†	65470	140580	206050	461120	9.81	21.07	69.12	0.22	0.48	1.56	NA
ESA	Angola	46940	93970	140920	346500	9.63	19.28	71.09	0.32	0.64	2.37	NA
ESA	Tanzania	34820	63460	98270	199860	11.68	21.28	67.04	0.31	0.56	1.75	NA
ESA	Uganda	31910	67130	99040	251960	9.09	19.13	71.78	0.22	0.46	1.74	NA
ESA	Kenya	26150	40330	66480	127580	13.47	20.78	65.74	0.33	0.51	1.63	NA
ESA	Mozambique	23900	37770	61670	123840	12.88	20.36	66.76	0.30	0.48	1.57	7.2 (2011)
ESA	Madagascar	19180	50820	70000	286820	5.38	14.24	80.38	0.12	0.31	1.76	NA
ESA	South Africa	16580	32740	49320	111620	10.30	20.34	69.35	0.23	0.46	1.58	4.5 (2016)
ESA	Sudan	13440	24160	37610	79390	11.49	20.65	67.86	0.27	0.49	1.62	NA
ESA	Malawi	13090	23140	36230	75770	11.69	20.66	67.65	0.28	0.49	1.62	NA
ESA	Zambia†	11690	32060	43750	126040	6.89	18.88	74.23	0.11	0.29	1.14	0.4 (2015)
ESA	Somalia†	10060	17870	27930	65790	10.74	19.07	70.19	0.25	0.45	1.65	NA
ESA	Zimbabwe*	8040	16390	24430	47430	11.19	22.80	66.00	0.22	0.44	1.27	NA
ESA	South Sudan†	6550	13390	19940	46120	9.92	20.27	69.82	0.24	0.48	1.66	NA
ESA	Rwanda	6050	14320	20370	59010	7.62	18.04	74.34	0.18	0.42	1.73	34 (2016)
ESA	Burundi	3270	11780	15050	57200	4.53	16.30	79.17	0.11	0.41	1.97	24.3 (2011)
ESA	Eritrea	1960	3560	5510	14920	9.57	17.41	73.02	0.22	0.41	1.71	1.5 (2011)
ESA	Botswana†	920	2520	3440	11160	6.27	17.27	76.46	0.16	0.44	1.94	NA
ESA	Namibia	820	2630	3460	12000	5.33	17.02	77.65	0.12	0.38	1.75	NA
ESA	Lesotho†	810	2120	2940	11390	5.68	14.82	79.50	0.12	0.32	1.73	NA
ESA	Swaziland†	550	1220	1770	5210	7.92	17.41	74.67	0.17	0.38	1.62	64.1 (2011)
ESA	Mauritius	340	990	1330	6140	4.57	13.23	82.19	0.11	0.31	1.91	5.5 (2015)
ESA	Djibouti	280	650	930	3240	6.64	15.64	77.72	0.11	0.25	1.24	21.3 (2018)
ESA	Comoros	200	770	960	4040	3.90	15.34	80.76	0.09	0.35	1.82	NA
ESA	Seychelles†	<100	<100	<100	460	4.31	12.22	83.47	0.10	0.29	2.01	0 (2016)
LAC	Brazil	38090	131420	169510	838900	3.78	13.03	83.19	0.07	0.23	1.46	2.1 (2016)
LAC	Mexico*	16520	70080	86600	478580	2.92	12.40	84.68	0.05	0.20	1.36	8.1 (2013)
LAC	Colombia	11100	33760	44860	187290	4.78	14.54	80.68	0.08	0.24	1.35	4.3 (2013)
LAC	Argentina	9130	22860	31990	101690	6.83	17.10	76.07	0.29	0.73	3.25	6.7 (2011)
LAC	Venezuela	8280	27300	35570	169260	4.04	13.33	82.63	0.07	0.24	1.48	NA
LAC	Guatemala	7960	23620	31570	105020	5.83	17.29	76.89	0.16	0.47	2.11	NA
LAC	Peru	7200	18660	25860	109670	5.31	13.77	80.92	0.10	0.26	1.50	NA
LAC	Haiti†	6870	19390	26260	99400	5.47	15.43	79.10	0.24	0.68	3.49	2.4 (2012)
LAC	Dominican Republic*	5620	16810	22430	111410	4.20	12.56	83.24	0.23	0.68	4.49	0.4 (2018)
LAC	Ecuador	4780	19610	24390	122230	3.26	13.38	83.37	0.05	0.22	1.39	1.5 (2017)
LAC	Bolivia	2940	11070	14010	62950	3.82	14.39	81.79	0.06	0.24	1.35	NA
LAC	Chile	2720	8340	11060	40340	5.29	16.23	78.48	0.09	0.27	1.32	0.6 (2012)
LAC	Cuba*	2670	10150	12830	71730	3.16	12.01	84.83	0.06	0.21	1.49	0 (2016)
LAC	Honduras	2470	7350	9820	32450	5.83	17.40	76.77	0.09	0.26	1.17	2.2 (2018)
LAC	Nicaragua	1790	5350	7130	27870	5.11	15.27	79.62	0.23	0.68	3.54	2.6 (2017)
LAC	El Salvador*	1520	4100	5620	22150	5.46	14.77	79.77	0.08	0.22	1.21	1.6 (2016)
LAC	Paraguay*	1330	4850	6170	24930	4.26	15.59	80.15	0.07	0.25	1.31	0.4 (2017)
LAC	Costa Rica†	1320	4700	6010	23670	4.43	15.82	79.75	0.07	0.25	1.28	NA
LAC	Jamaica	830	2740	3570	17150	4.00	13.23	82.77	0.06	0.21	1.30	0 (2017)
LAC	Panama†	760	2460	3220	14130	4.37	14.17	81.46	0.07	0.22	1.28	0.4 (2018)
LAC	Uruguay*	630	2150	2780	13500	3.87	13.22	82.91	0.08	0.26	1.62	NA
LAC	Guyana	490	1400	1880	5910	6.26	17.90	75.84	0.24	0.69	2.93	5.8 (2014)
LAC	Trinidad & Tobago	480	1650	2130	14500	2.86	9.94	87.20	0.13	0.47	4.11	NA
LAC	Suriname	330	1020	1350	5690	4.68	14.47	80.85	0.22	0.68	3.80	9.5 (2018)
LAC	Bahamas	250	700	940	3800	5.20	14.67	80.13	0.23	0.64	3.52	NA
LAC	Barbados	160	450	610	2870	4.71	12.94	82.35	0.25	0.68	4.31	NA
LAC	Belize	<100	310	400	1780	4.32	14.17	81.52	0.19	0.61	3.52	NA
LAC	St. Lucia*	<100	270	340	1380	4.20	15.50	80.30	0.06	0.24	1.22	0 (2012)
LAC	St. Vincent & Grenadines	<100	200	270	1020	5.36	15.43	79.20	0.24	0.70	3.62	NA
LAC	Grenada	<100	180	240	1070	4.38	13.79	81.83	0.20	0.64	3.81	NA
LAC	Antigua & Barbuda	<100	160	220	970	4.58	13.50	81.92	0.21	0.62	3.79	NA
MENA	Iran	12500	51060	63560	323130	3.23	13.20	83.56	0.05	0.20	1.26	2.3 (2015)
MENA	Egypt	9650	30060	39710	252920	3.30	10.27	86.43	0.04	0.13	1.09	0.7 (2015)
MENA	Morocco*	7500	19900	27400	117220	5.19	13.76	81.06	0.08	0.21	1.21	0 (2016)
MENA	Algeria	7400	20390	27790	141870	4.36	12.02	83.62	0.07	0.18	1.28	3.5 (2017)
MENA	Iraq	3060	17840	20900	131060	2.01	11.74	86.25	0.03	0.17	1.26	NA
MENA	Yemen	2360	13720	16070	97500	2.07	12.08	85.85	0.03	0.17	1.24	NA
MENA	Tunisia	1870	5450	7320	38890	4.05	11.79	84.16	0.06	0.18	1.29	0 (2014)
MENA	Syria	1270	8000	9260	103260	1.12	7.11	91.77	0.01	0.09	1.20	NA
MENA	Israel	1180	3360	4540	21730	4.50	12.78	82.72	0.06	0.17	1.13	NA
MENA	Saudi Arabia	1010	6580	7590	58890	1.52	9.89	88.59	0.02	0.14	1.22	NA
MENA	Libya	730	4170	4900	33500	1.89	10.86	87.24	0.03	0.15	1.21	NA
MENA	Jordan	420	2460	2880	16300	2.20	12.83	84.97	0.03	0.19	1.24	NA
MENA	Palestinian Territories	370	2290	2660	23190	1.44	8.86	89.70	0.02	0.11	1.15	NA
MENA	Lebanon	360	2390	2740	23000	1.38	9.27	89.35	0.02	0.13	1.23	NA
MENA	Kuwait	180	1330	1510	21930	0.77	5.68	93.56	0.01	0.06	1.07	NA
MENA	United Arab Emirates	140	940	1090	12240	1.08	7.08	91.85	0.01	0.09	1.23	NA
MENA	Bahrain	120	690	810	11890	0.91	5.45	93.64	0.01	0.07	1.26	NA
MENA	Oman	<100	320	400	4210	1.57	7.01	91.42	0.02	0.09	1.17	NA
MENA	Qatar	<100	310	360	5140	0.90	5.56	93.54	0.01	0.07	1.13	NA
NA	United States	49410	127990	177400	757000	5.29	13.70	81.01	0.07	0.17	1.00	NA
NA	Canada	3590	12350	15950	89110	3.42	11.76	84.82	0.04	0.15	1.05	NA
SA	India	157410	534210	691620	3046660	4.21	14.29	81.50	0.04	0.15	0.84	1.2 (2017)
SA	Bangladesh*	45770	75610	121380	338320	9.96	16.45	73.60	0.10	0.16	0.72	0.1 (2016)
SA	Pakistan*	25320	94840	120160	484430	4.19	15.69	80.12	0.04	0.17	0.85	3.8 (2016)
SA	Afghanistan†	9530	22790	32320	74650	8.91	21.30	69.79	0.10	0.24	0.78	0.3 (2012)
SA	Nepal*	6250	16140	22390	66210	7.05	18.22	74.73	0.07	0.18	0.72	NA
SA	Sri Lanka*	4210	9210	13420	52660	6.37	13.94	79.69	0.08	0.17	0.98	0 (2016)
SA	Bhutan	90	300	390	1640	4.48	14.92	80.60	0.04	0.14	0.78	NA
SA	Maldives	20	110	130	970	2.07	9.70	88.24	0.02	0.09	0.84	NA
WCA	Nigeria	87130	171760	258880	620220	9.91	19.54	70.55	0.18	0.35	1.26	9.8 (2015)
WCA	Congo-Kinshasa*	70640	118460	189100	389800	12.20	20.46	67.33	0.34	0.57	1.87	4.5 (2012)
WCA	Niger	19620	26000	45620	74410	16.35	21.66	61.99	0.37	0.49	1.40	13.9 (2015)
WCA	Côte d’Ivoire	14020	23370	37390	74570	12.52	20.87	66.60	0.21	0.35	1.12	2.4 (2014)
WCA	Cameroon	13740	22290	36020	77840	12.06	19.57	68.36	0.21	0.33	1.16	27.5 (2012)
WCA	Mali†	12570	18350	30920	57120	14.28	20.84	64.88	0.27	0.39	1.21	NA
WCA	Ghana	11760	26680	38440	108290	8.01	18.19	73.80	0.15	0.34	1.37	3.4 (2016)
WCA	Chad	10340	15460	25800	44840	14.64	21.88	63.48	0.27	0.40	1.15	19.6 (2011)
WCA	Burkina Faso	9130	18140	27270	62570	10.16	20.19	69.64	0.18	0.36	1.23	3.6 (2017)
WCA	Guinea	7510	12290	19800	38180	12.95	21.20	65.86	0.22	0.37	1.14	10.7 (2017)
WCA	Benin	5100	10320	15420	36610	9.80	19.84	70.36	0.17	0.35	1.23	2.9 (2017)
WCA	Senegal	4320	6800	11110	22720	12.76	20.09	67.15	0.21	0.33	1.12	NA
WCA	Sierra Leone	4260	12920	17180	57300	5.72	17.34	76.94	0.10	0.30	1.35	3.3 (2016)
WCA	Togo	3540	6730	10270	25270	9.95	18.94	71.12	0.17	0.32	1.20	5.3 (2017)
WCA	Central African Republic	3380	4870	8250	12970	15.94	22.95	61.11	0.28	0.41	1.09	14.6 (2014)
WCA	Liberia	3370	6740	10110	26410	9.22	18.45	72.32	0.29	0.58	2.26	0 (2014)
WCA	Congo-Brazzaville	3050	4570	7620	16560	12.62	18.90	68.48	0.22	0.33	1.21	3.5 (2018)
WCA	Mauritania	2910	4240	7150	14200	13.64	19.85	66.51	0.23	0.34	1.12	NA
WCA	Gabon	890	1520	2410	6950	9.55	16.24	74.22	0.16	0.27	1.23	NA
WCA	Guinea-Bissau	730	1610	2340	6250	8.48	18.74	72.78	0.14	0.32	1.23	22.2 (2011)
WCA	Gambia	680	1980	2660	7740	6.56	19.03	74.41	0.11	0.33	1.28	8.3 (2018)
WCA	Equatorial Guinea	550	990	1540	3760	10.40	18.70	70.91	0.17	0.31	1.17	NA
WCA	Cape Verde	140	340	480	1870	5.95	14.40	79.66	0.09	0.22	1.23	3.9 (2013)
WCA	São Tomé & Príncipe	100	180	270	650	10.28	19.12	70.60	0.18	0.33	1.21	NA
WE	Germany	8920	22220	31140	145100	5.06	12.61	82.33	0.06	0.15	0.97	NA
WE	United Kingdom	8670	25600	34270	181490	4.02	11.87	84.12	0.05	0.15	1.09	0 (2013)
WE	France	6180	20160	26340	147870	3.55	11.57	84.88	0.04	0.15	1.07	NA
WE	Italy	5190	16070	21260	136220	3.30	10.20	86.50	0.04	0.13	1.12	NA
WE	Spain	2190	9720	11910	109060	1.81	8.04	90.15	0.02	0.10	1.08	2.2 (2015)
WE	Netherlands	1990	5570	7550	36090	4.55	12.75	82.70	0.06	0.15	1.00	NA
WE	Poland	1860	8840	10700	97560	1.72	8.17	90.12	0.02	0.10	1.13	NA
WE	Sweden	1200	2870	4070	21480	4.68	11.24	84.08	0.06	0.13	1.00	NA
WE	Belgium	1140	2750	3900	27530	3.63	8.77	87.60	0.05	0.12	1.17	0.1 (2013)
WE	Austria	1130	3430	4560	25240	3.79	11.51	84.71	0.05	0.14	1.02	1.3 (2015)
WE	Czechia	940	2640	3590	19520	4.09	11.44	84.48	0.05	0.14	1.01	NA
WE	Portugal	850	2730	3580	24070	3.09	9.86	87.05	0.04	0.12	1.10	NA
WE	Norway	750	1830	2580	11050	5.50	13.43	81.07	0.06	0.15	0.91	NA
WE	Denmark	730	2750	3480	23690	2.70	10.12	87.19	0.03	0.12	1.07	9.1 (2011)
WE	Hungary	680	1960	2640	11840	4.71	13.53	81.75	0.05	0.16	0.95	NA
WE	Switzerland	600	2300	2900	19300	2.68	10.36	86.96	0.03	0.12	1.02	NA
WE	Finland	550	1700	2240	12040	3.83	11.88	84.29	0.05	0.15	1.06	NA
WE	Ireland	410	1350	1760	11000	3.22	10.54	86.24	0.04	0.11	0.94	NA
WE	Greece	370	2050	2420	24460	1.38	7.64	90.98	0.02	0.09	1.13	NA
WE	Slovakia	240	1180	1410	13910	1.54	7.69	90.77	0.02	0.09	1.09	NA
WE	Slovenia	140	640	780	6650	1.88	8.61	89.51	0.03	0.12	1.22	NA
WE	Lithuania	140	360	500	4690	2.67	6.94	90.39	0.04	0.09	1.21	NA
WE	Latvia	130	480	610	4650	2.51	9.04	88.45	0.03	0.11	1.11	NA
WE	Estonia	100	290	390	3170	2.67	8.25	89.08	0.03	0.10	1.13	NA
WE	Cyprus	<100	340	390	3020	1.64	9.90	88.46	0.02	0.11	1.00	NA
WE	Luxembourg	<100	170	210	1370	2.67	10.45	86.88	0.03	0.11	0.91	NA
WE	Iceland	<100	110	150	710	4.75	12.84	82.41	0.05	0.14	0.91	NA
WE	Malta	<100	120	150	990	2.76	10.47	86.77	0.03	0.12	1.02	NA

*Based on adult data assessed as nationally adequate;

^†^ Based on adult data assessed as nationally inadequate but regionally adequate;

all other countries have either no documented size estimates and/or used inadequate methods.

**Fig 1:**
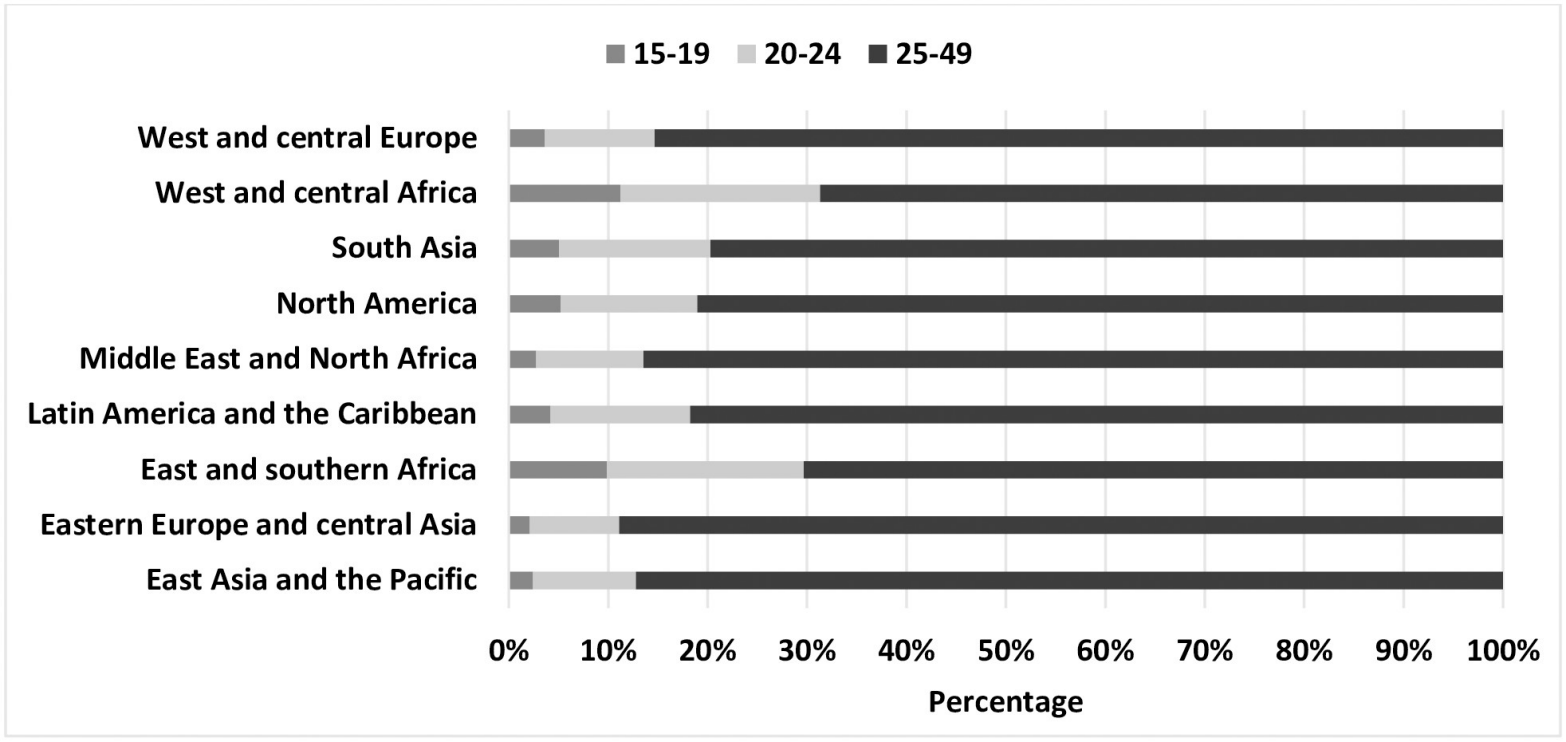
Distribution of population estimates of men who have sex with men by age group and UNICEF region.

**Fig 2:**
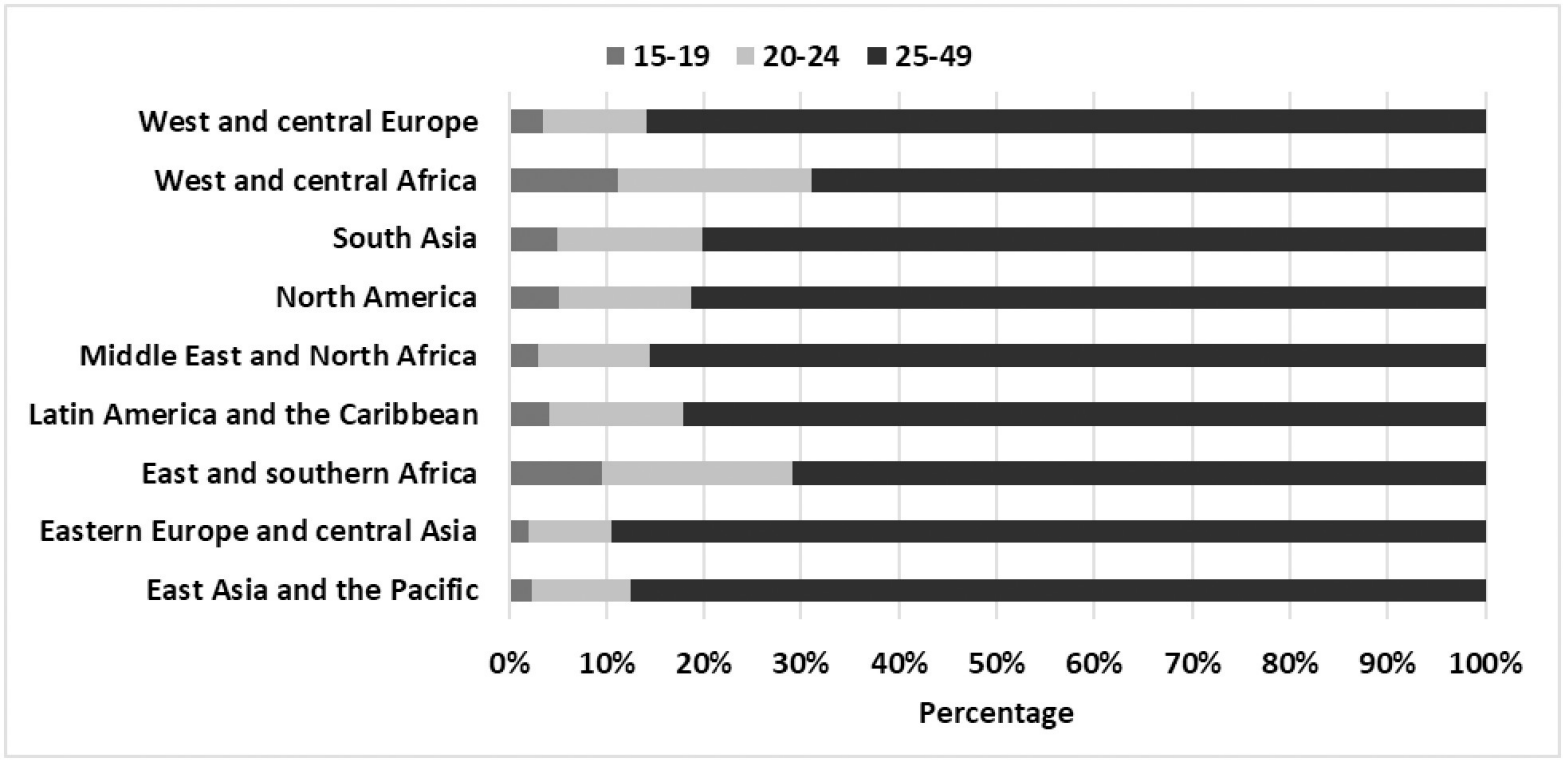
Distribution of population size estimates of female sex workers by age group and UNICEF.

Differences in sexual initiation led to different distributions of young men who have sex with men across geographic regions. Globally, an estimated 17% of men who have sex with men were between the ages of 15–24; 4% were adolescents. The averaged proportions of adolescent men who have sex with men (among all men who have sex with men) ranged from 2% in East Asia and the Pacific and eastern Europe and central Asia to 10% in eastern and southern Africa (Fig 1). The average proportion of young (20 to 24 years) men who have sex with men among all estimated men who have sex with men ranged from 9% in eastern Europe and central Asia to 20% in eastern and southern and western and central Africa regions. Among an estimated 69.5 million men who have sex with men aged 15–49, an estimated 12 million are under the age of 24 years, of whom 3 million are adolescents. Of all estimated females who sell sex in their respective regions, adolescent females who sell sex comprised from 2% in East Asia and the Pacific and eastern Europe and central Asia to 11% in West and central Africa and young females who sell sex comprised from 9% in eastern Europe and central Asia to 20% in West and central Africa (Fig 2). Adolescent girls who sell sex were estimated at 1.4 million (note: The United Nations does not recognize “sex workers” under the age of 18 and considers girls under age 18 selling sex as exploited youth. We are currently unable to estimate such exploited youth under 18) and there were an estimated 3.7 million 20-24-year-old females engaged in sex work. Limited HIV prevalence data indicates a sizable proportion of adolescent and young boys who have sex with males and females who sell sex are living with HIV.

## Discussion

These population size estimates of adolescent and young men who have sex with men and females who sell sex in 184 countries suggest there are roughly 17 million adolescent and young males who have sex with males and females who sell sex. UNAIDS estimates approximately 10% of new infections among people 15–49 years old occur among young key populations (Unpublished data, personal report, last author). These data can be used to shape and improve the response to the HIV epidemic in these countries. The countries with the highest proportions of men who have sex with men and females who sell sex comprising the equivalent population, (i.e., general male population for men who have sex with men and general female population for females who sell sex) were often found in less populous countries. The highest proportions of adolescent and young men who have sex with men varied widely among countries; the highest proportions of adolescent females who sell sex are found in sub–Saharan Africa, and the highest proportions of young females who sell sex are in Latin America and the Caribbean region.

There are still limited data available on HIV prevalence among adolescent and young key populations. HIV prevalence among men who have sex with men and females who sell sex under the age of 25 exceeded 20% in many reporting countries. UNAIDS estimates that about 70% of HIV infections among males 15–24 occurred among men who have sex with men, transmen and male sex workers and 25% of new HIV infections among adolescent and young females were among females who sell sex or transwomen (Unpublished data, personal report, last author). Surveys that include HIV testing often omit people who are under the age 18 for legal or ethical reasons. Nonetheless, HIV prevalence among young people, under 25 years, indicate that HIV acquisition is appreciable in this age group, warranting attention from HIV prevention and treatment services. Currently, most countries require that a parent accompany a minor to get HIV testing and the few countries that allow a minor to receive HIV testing, require parental consent for positive minors to get care and treatment.

The size estimates presented here provide national programmes with data to plan the scale of youth-friendly services. These data also provide evidence for the need to increase flexibility with age limits for HIV and sexual/reproductive health services and treatment, especially for the most vulnerable adolescent populations. This publication offers an initial step in producing usable estimates of the size of adolescent and young vulnerable populations; however, several inconsistencies and limitations in these findings warrant caution in their use. WHO, UNAIDS and GFATM jointly assessed size estimates available by the end of 2019, only 41 countries for males who have sex with males and 19 countries for females who sell sex were deemed to have “nationally adequate” estimates, meaning that they are empirically derived and/or reflect geographic coverage of >50% of the population [1]. Given the importance of size estimates among adolescent and young key populations, as well as adult key populations, more attention is needed for countries to collect the data, formulate their estimates, and clearly describe their methods.

Our estimates are probably non-differentially biased across the breadth of countries but may over- or underestimate in any given country, especially for adolescents since data were adjusted based on the average age at sexual debut of general population adolescents. However, if the bias is directional, it is probably toward underestimation, as sexual debut is likely underreported. Men who have sex with men and females who sell sex may initiate sex at different ages from the rest of their age cohort. Indeed, we were not able to estimate the population sizes of adolescent and young people who inject drugs due to the lack of data on age of first injection. For countries and regions where surveys on age of first sex were not available, we extrapolated the estimate based on the global and regional trends using only the neighboring structure of the countries and thus did not capture local variations in the age-at-initiation. Future studies could include covariates that might be relevant to culture norms, such as economic status and religion, to improve the extrapolated estimates of the age-at-initiation of sex. Some estimates are likely to be biased by poor implementation of methods, levels of stigma and discrimination in different contexts, and government pressure to minimize the mere existence of these populations [26]. The year in which the original population size estimates were calculated may not correspond with the year (2019) of the general population data. However, reported size estimations were conducted within the past five years, thereby reducing the impact of population fluctuations on the final men who have sex with men and females who sell sex size estimates. The most appropriate estimate of the proportion of adolescents and young key populations would be to use the proportions sampled directly from the survey source, assuming a probability-based sampling method was used. However, many countries do not report these age breakdowns (i.e., 15 to 19, 20 to 24) and many surveys do not sample men who have sex with men or female who sell sex under the age of 18. Furthermore, age-specific adjustments to the proportion of the general population that belongs to each population group would be possible if data were available from men who have sex with men and females who sell sex surveys on the age at sexual debut (or drug injection initiation for young people who inject drugs). We recommend that countries: 1) include programming for adolescents and young men who have sex with men and females who sell sex in their national HIV response; 2) present disaggregated age in surveys, and 3) calculate population size estimates findings by 15 to 19 years and 20 to 24 years[5, 27]. Such surveys and studies should also collect and publish data on age-at-sexual debut. Ideally, data from surveys of key populations, much like the demographic and health survey data, should be made available for others to conduct further analyses.

Finally, a uniform eligibility criterion is needed. Size estimates vary depending on if the definition of men who have sex with men as “ever having anal sex,” “having anal sex in the past one month” or “ever engaged in same sex sexual activity [28].” For the purposes of HIV IBBS surveys, there are recommendations to define men who have sex with men as having anal sex with a man and females who sell sex as having anal or vaginal sex with in the past six [29, 30]. Additionally, transgender women, given their unique risk, stigma and health needs, should be surveyed and counted apart from men who have sex with men. These differences combined with different years or sub-regions of data collection make interpretation challenging.

## Conclusions

Despite the limitations, these findings provide a foundation on which to improve national HIV responses and the general health needs of highly vulnerable young people. Additionally, high quality anthropological and sociological research, as well as deeper secondary analysis from biological behavioral surveillance surveys of these populations, can further enrich understanding and provision of needed services. In addition, these findings show the need for adolescents to have access to age-appropriate health care, including HIV testing, care, and treatment [28]. Adolescent key populations face policy and legal barriers related to age of consent, with third party authorization requirements, which prevent access to health services related to HIV and other sexually transmitted infections and harm reduction. Essential HIV services for adolescent and young key populations will increase the chances that they can protect themselves and receive interventions before they contract HIV. Given the substantial numbers of adolescents practicing high risk behaviors and at risk for HIV exposure, services must find creative ways to engage adolescents and young people from the community. These possibly lifesaving services can be planned and funded based on the estimates presented in this paper, to provide essential, support to an estimated 4 million adolescents and 17 million young key populations around the world. We strongly encourage countries without adequate or any size estimations of these populations to produce them.
